# Comparison between single anterior and single posterior approaches of debridement interbody fusion and fixation for the treatment of mono-segment lumbar spine tuberculosis

**DOI:** 10.1007/s00402-021-03955-4

**Published:** 2021-05-22

**Authors:** Hangli Wu, Yaqing Cui, Liqun Gong, Jun Liu, Yayi Fan, Yongchun Zhou, Weiwei Li

**Affiliations:** 1 Department of Orthopedic, Shaanxi Provincial People’s Hospital, Xi’an, 710068 Shaanxi China; 2 Department of Plastic and Reconstructive Surgery, Shaanxi Provincial People’s Hospital, Xi’an, 710068 Shaanxi China

**Keywords:** Spinal tuberculosis, Anterior, Posterior, Mono-segment, Lumbar

## Abstract

**Purpose:**

To compare the efficacy of single anterior and single posterior approach of debridement, interbody fusion, and fixation for the treatment of mono-segment lumbar spine tuberculosis (TB) patients.

**Methods:**

Eighty-seven patients with mono-segment lumbar TB who underwent debridement, interbody fusion, and fixation through either single anterior (Group A) or single posterior approach (Group B) from January 2007 to January 2017 were enrolled in this study. The duration of the operation, blood loss, complication rate, visual analog scale (VAS), Oswestry disability index (ODI), Frankel scale, erythrocyte sedimentation rate (ESR), C-reactive protein (CRP), kyphosis angle, correction rate, correction loss, and time taken for bone graft fusion were compared between the groups.

**Results:**

The average period of follow-up was 34.3 ± 9.5 months (24–56 months). No significant differences were observed between patients in Group A and patients in Group B in terms of gender, age, body mass index (BMI), duration of illness and preoperative evaluative indices (*P* > 0.05). The mean operation time and blood loss was significantly higher in Group A (*P* = 0.000), along with a slightly higher rate of complications compared with Group B (*P* = 0.848). The VAS, ODI and Frankel scale scores showed significant improvement in both groups (*P* = 0.000), along with the ESR, CRP and kyphosis indices (*P* = 0.000), which were similar in both groups at the final follow-up.

**Conclusion:**

Both single anterior and single posterior approaches of debridement, interbody fusion and fixation are effective for mono-segment lumbar TB patients, although the single posterior approach is of a shorter duration and results in less blood loss.

## Introduction

Spinal tuberculosis (TB) is a chronic infection of the spine that causes pain, kyphotic deformity, and disability. Spinal TB accounts for a large proportion of extrapulmonary TB cases and is the most common form of bone and joint TB. Despite considerable efforts made by the WHO during the past decade to prevent and control TB, spinal TB is still prevalent in China, India, and other developing and undeveloped countries [1]. In addition, sporadic cases have also been reported in some developed countries [2].

Most spinal TB patients can be cured using regular anti-TB drug chemotherapy along with bed rest and nutritional supplements [3]. However, anti-TB drugs are not effective against kyphosis deformity. Rajasekaran et al. [4] found that kyphosis deformity progressed in 39% of the pediatric patients with spinal TB who received non-surgical treatment. Cold abscess sinus formation, spinal disability, neurological deficits, and severe kyphosis deformity are currently recognized as indications for surgical intervention for spinal TB [5]. The objectives of surgery are debridement, neurological decompression, and re-stabilization of the spine. An anterior surgical approach was initially developed by Hodgson and Stock [6] and has been known as the Hong Kong technique. The single posterior approach of debridement, interbody fusion and fixation was subsequently developed for patients with lumbar TB [7], who account for a considerable proportion of spinal TB cases. However, only a few studies have compared the therapeutic efficacy of single anterior and single posterior approach of debridement, interbody fusion, and fixation. Therefore, the aim of this study is to compare the therapeutic efficacy of both approaches for mono-segmental lumbar TB patients.

## Materials and methods

### Patient population

The inclusion criteria were as follows: (1) confirmed diagnosis of lumbar spinal TB, (2) lesion was limited to one disc and two adjacent vertebral bodies, (3) elective surgical debridement, interbody fusion, and fixation via either a single anterior or a single posterior approach, and (4) follow-up duration of at least 24 months. Patients with systemic TB, a history of abdominal or lumbar surgery and inability to sustain long-term and effective anti-TB drug treatment due to damaged hepatic function were excluded. Among the initial cohort of 167 patients with lumbar spinal TB, 38 were reluctant to undergo surgery and 42 did not meet the inclusion criteria. Finally, 87 cases that met the inclusion criteria and provided consent for surgery were enrolled. The diagnosis was confirmed through MTB culture or pathological examination. Routine blood test, erythrocyte sedimentation rate (ESR), C-reactive protein (CRP) and T cell spot test for TB infection (T-SPOT.TB) were performed on all patients. X-ray, CT, and MRI examinations were pre-operatively performed to develop a diagnosis and treatment plan. All patients manifested varying degrees of back pain and fatigue, although only 33 cases (37.9%) presented with typical symptoms of TB, such as low-grade fever in the evenings, night sweats and fatigue. All participants provided written informed consent to participate in the study.

### Preoperative preparation

The patients received 2–4 weeks of HREZ standard chemotherapy regimen (including isoniazid, rifampicin, ethambutol, and pyrazinamide) before the operation. Sufficient nutritional supplementation was also provided.

### Operative techniques

**Fig. 1:**
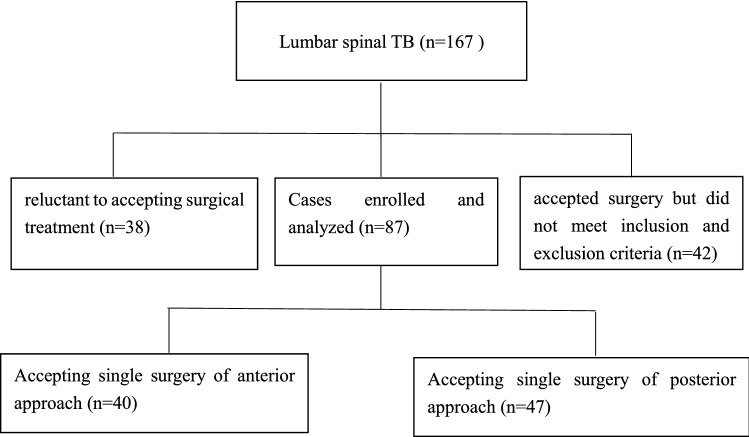
Flow chart for patients enrolled

Single anterior approach (Group A): An oblique incision was made above the groin, and the muscles were bluntly separated layer by layer while protecting the retroperitoneum and intra-abdominal organs. The psoas muscle abscess was cleared by rinsing and wiping it with a wet gauze, and the necrotic disc, sequestrum, diseased granulation tissue and pus were carefully removed. A three-sided cortical iliac was obtained from the same incision, and an interbody bone graft was performed after debridement. A vertebral screw-rod system or a screw-plate was used for vertebral fixation (Fig. 1).

**Fig. 2:**
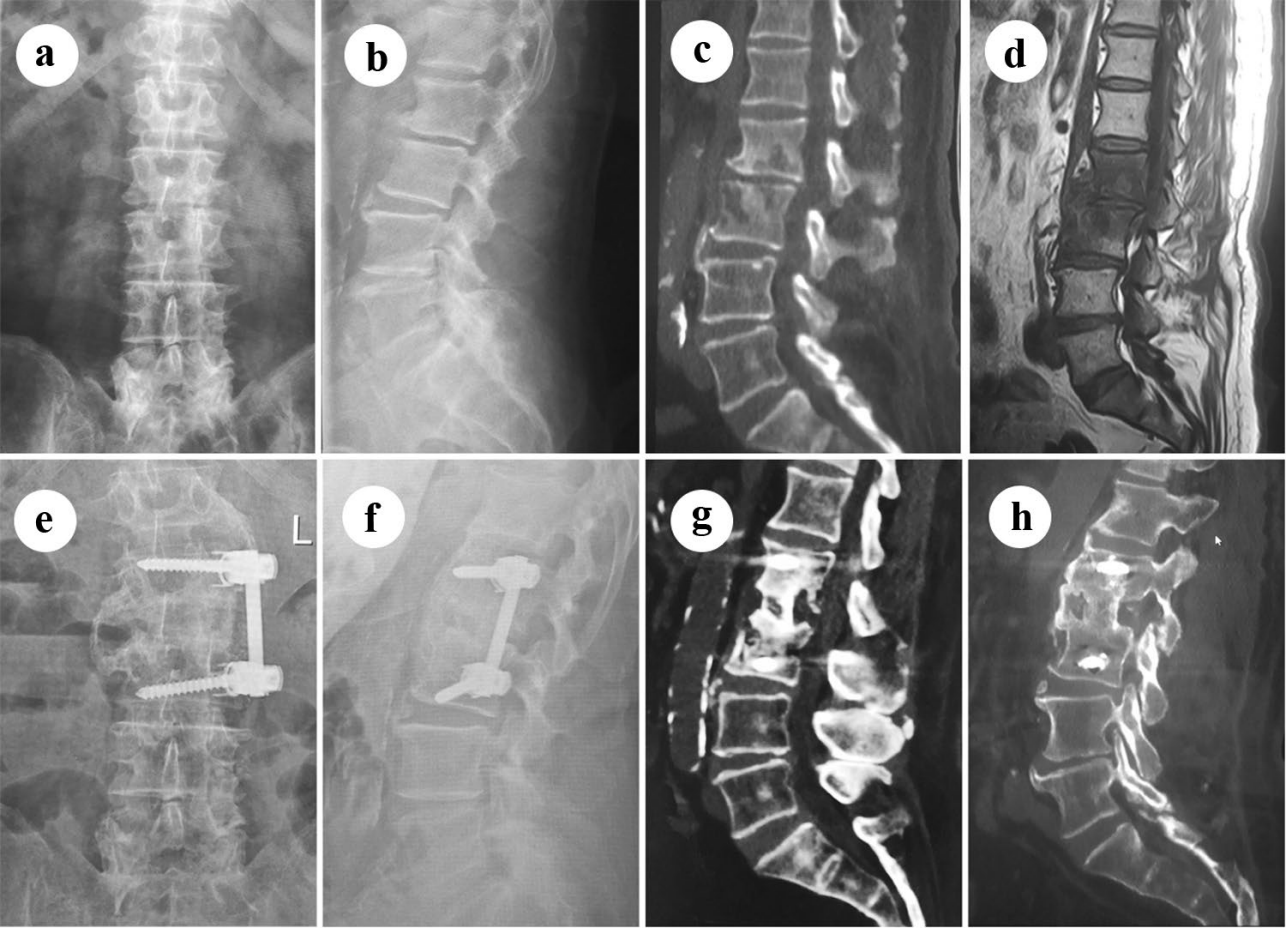
A 73-year-old male patient with L2–3 mono-segmental spinal TB, who complained of severe back pain for 7 months, received single anterior debridement, interbody fusion and vertebral fixation. **a**, **b** Preoperative X-ray of AP and lateral images show lumbar degeneration and loss of L2-3 intervertebral height. **c**, **d** Preoperative CT and MRI images show the destruction of vertebral bodies and disc. **e**, **f** Postoperative X-ray of AP and lateral images show good position of vertebral screw fixation. **g**, **h** Postoperative CT images show solid interbody fusion had been achieved at the postoperative 24th month

Single posterior approach (Group B): A midline incision was made, and the paravertebral muscle was dissected subperiosteally to expose the lamina and facet joints within the affected region. Pedicle screws were used in the normal vertebral body and occasionally in the affected vertebral body. The fixation range was minimized as much as possible. Total laminectomy and partial facet joint resection were performed, followed by debridement under the protection of the dura and nerve root. The necrotic disc, caseous granulated tissue and dead bones were removed using different curettes. Non-structural interbody fusion was implemented using bone particles obtained from the normal lamina and facet joints. Finally, two good bended rods were placed on either side to fix the affected area (Fig. 2).

### Postoperative management

**Fig. 3:**
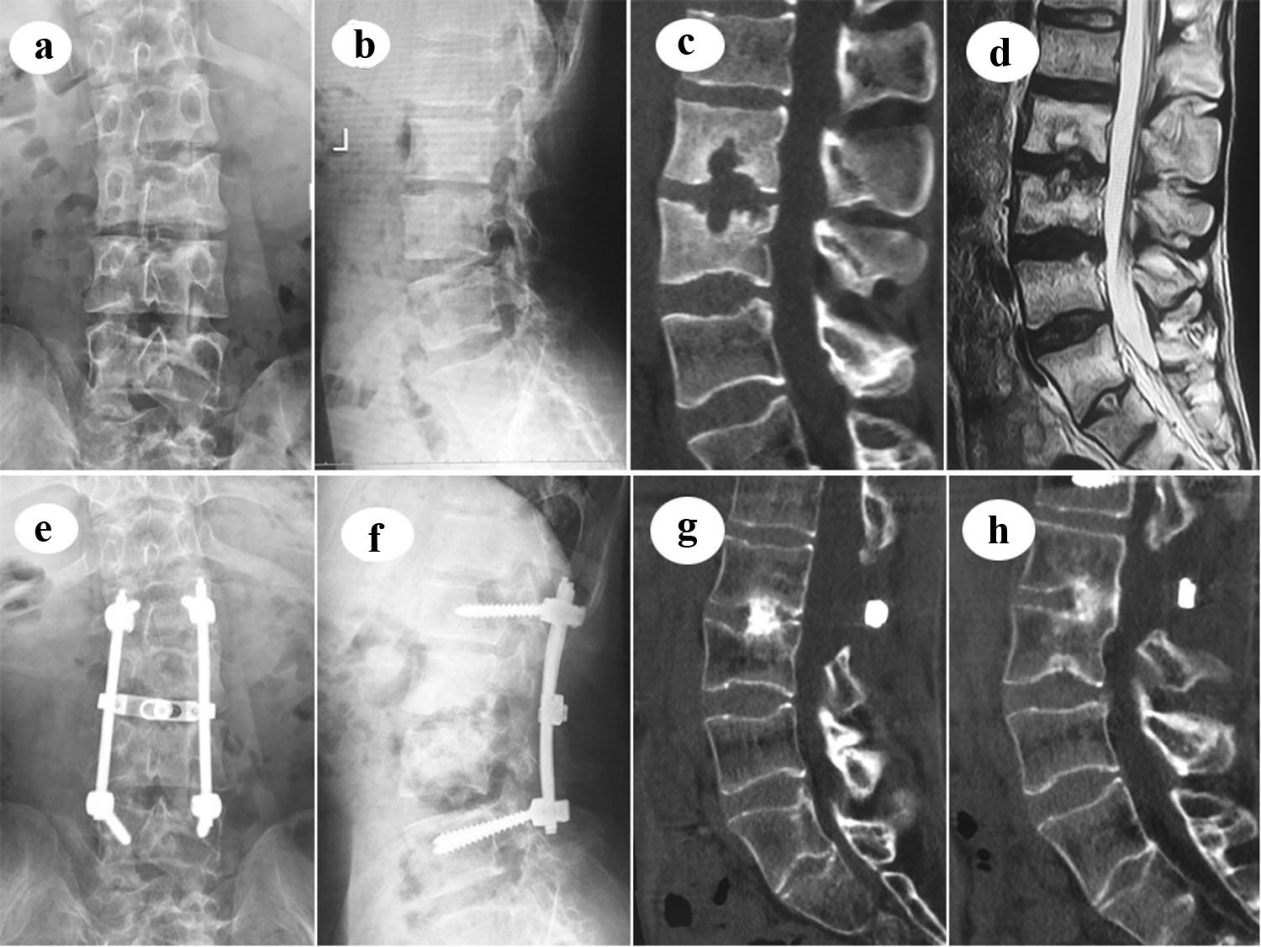
A 29-year-old male patient with L2–3 mono-segmental spinal TB, who complained of severe low back pain for 3 months, received single posterior debridement, interbody fusion and pedicle fixation. **a**, **b** Preoperative X-ray of AP and lateral images show roughly normal radiological presentation. **c**, **d** Preoperative CT and MRI images show the destruction of vertebral bodies and disc. **e**, **f** Postoperative X-ray of AP and lateral images show good position of pedicle screw fixation. **g**, **h** Postoperative CT images show solid interbody fusion had been achieved at the postoperative 24th month

Antibiotics were administered for at least 24 h to prevent infection, while hepato-protective drugs were administered prophylactically. Partial weight-bearing ambulation with a thoracolumbar brace was recommended to be used from the 6th week post-operation. The minimum duration of HREZ standard chemotherapy administration was 12 months (Fig. 3).

### Evaluation indexes

The duration of operation, blood loss, complication rate, visual analog scale (VAS), Oswestry disability index (ODI), Frankel scale, ESR, CRP level, kyphosis Cobb’s angle, kyphosis correction rate, kyphosis correction loss and duration of bone graft fusion were compared between the two groups.

### Statistical analysis

Statistical analyses were performed using SPSS version 22.0 software (SPSS Inc., Chicago, IL, USA). Independent sample *t*-test, Chi-squared and or Wilcoxon signed rank test were conducted. A *P* value of < 0.05 was considered to indicate statistical significance.

## Results

### Demographic data

Group A included 24 males and 16 females of a mean age of 52.2 ± 13.4 years (24–76 years), mean body mass index (BMI) of 20.7 ± 2.6 kg/m^2^ (16–28 kg/m^2^), and mean illness duration of 5.3 ± 1.6 months (1–8 months). Group B included 31 males and 16 females of a mean age 50.3 ± 9.9 years (25–74 years), mean BMI of 21.4 ± 2.9 kg/m^2^ (16–30 kg/m^2^), and mean illness duration of 5.0 ± 1.2 months (1–8 months). Both groups were similar in terms of gender, age, and BMI (*P* > 0.05). The patients were followed-up for 24–56 months and the mean follow-up period was 34.3 ± 9.5 months

### Surgical data

The mean duration of the operation and mean intra-operative blood loss were significantly lower in Group B compared with that of Group A (*P* < 0.05; Table 1). No intra-operative neurological, vascular, urethral, or visceral injuries had occurred in either group, although two cases of dura tear were reported from Group B. No cases of TB relapse were recorded in Group A, while one case of relapse was reported from Group B, which was cured using anterior debridement surgery three months after the first surgery. The surgical complication rate in Group A was insignificantly higher than that of Group B (*P* > 0.05; Table 1).

**Table 1:** Evaluation indexes comparison between Group A and Group B

Evaluation indexes	Group A (*N* = 40)	Group B (*N* = 47)	*P* value
Operation time (min)	218.5 ± 15.5*	163.8 ± 13.3*	0.000
Blood loss (ml)	663.8 ± 82.2*	509.8 ± 72.1*	0.000
Rate of surgical complications (%)	7.5% (3/40)	6.4% (3/47)	0.848
Time of bone graft fusion (months)	6.5 ± 0.8	6.6 ± 0.7	0.794
Kyphosis correction rate (%)	84.2 ± 14.7	80.2 ± 20.5	0.310
Correction loss rate (%)	6.1 ± 6.9	5.5 ± 6.5	0.696

Data are presented as *n* (%) or mean ± standard deviation (range)

*: *P* < 0.05, the difference between Group A and Group B was significant

### Lumbar symptoms and function

No significant differences were observed between the pre-operative VAS and ODI values of the two groups (*P* > 0.05) and significant improvement was observed in both groups after the operation (*P* < 0.05). However, the post-operative and follow-up VAS and ODI values were similar in both groups (*P* > 0.05; Table 2).

**Table 2:** Comparison of VAS, ODI, ESR, CRP, and kyphosis angle between Group A and Group B

Schedule	Group A		Group B	
	Pre-op	FFU	Pre-op	FFU
VAS	4.2 ± 1.0	0.5 ± 0.6*	4.4 ± 1.0	0.4 ± 0.5*
ODI (%)	28.7 ± 10.0	7.4 ± 2.6*	30.9 ± 9.0	8.3 ± 4.6*
ESR (mm/h)	58.7 ± 9.4	13.0 ± 2.0*	57.1 ± 6.7	12.8 ± 2.0*
CRP (mg/L)	58.7 ± 13.3	2.8 ± 0.9*	56.4 ± 15.0	2.7 ± 1.0*

*Pre-op* pre-operation, *Post-op* post-operation, *FFU* final follow-up

*: *P* < 0.05, compared with pre-op indexes

### Laboratory test

The pre-operative ESR and CRP values were similar in both groups (*P* > 0.05) and decreased to baseline levels in Group A and B patients within 6 months post-operation. No significant differences were detected in any of the indices in both groups at the postoperative and final follow-up timepoints (*P* > 0.05; Table 2).

### Radiological evaluation

The pre-operative kyphosis Cobb’s angles were similar in both groups (*P* > 0.05), and patients in both groups showed significant kyphosis correction (*P* < 0.05). No significant differences were observed in the post-operative kyphosis Cobb’s angles, kyphosis correction rate and correction loss (*P* > 0.05) as well as in the interbody fusion duration between the two groups (*P* > 0.05; Table 3).

**Table 3:** Comparison of radiological indexes between Group A and Group B

Schedule	Group A		Group B	
	Pre-op	FFU	Pre-op	FFU
Kyphosis angle (°)	11.2 ± 2.6	2.2 ± 2.0*	12.1 ± 2.8	1.8 ± 1.7*
Time of bone graft fusion (M)	6.5 ± 0.8		6.6 ± 0.7	
Kyphosis correction rate (%)	84.2 ± 14.7		80.2 ± 20.5	
Correction loss rate (%)	6.1 ± 6.9		5.5 ± 6.5	

*Pre-op* pre-operation, *Post-op* post-operation, *FFU* final follow-up

*: *P* < 0.05, the difference of kyphosis angle between pre-op and FFU was significant in both Group A and Group B

### Neurological deficit assessment

As shown in Table 4, no significant difference was observed in the Frankel scale scores between the two groups (*P* > 0.05; Table 4).

**Table 4:** Comparison of Frankel grade between Group A and Group B

Preoperative		Final follow-up				
		A	B	C	D	E
D	32	0	0	0	2	30
E	8	0	0	0	0	8
D	37	0	0	0	3	34
E	10	0	0	0	0	10

## Discussion

The aim of this study was to compare the therapeutic efficacy of a single anterior approach and single posterior approach for the debridement, interbody fusion, and fixation for patients with mono-segment lumbar TB. Both techniques performed well in terms of multiple indices, and achieved pain relief, lumbar function recovery, neurological deficit improvement, lesion healing, and the restoration and maintenance of normal lumbar alignment to a similar extent. Furthermore, both the anterior and posterior approaches were associated with a low risk of surgical complications. However, single posterior surgery required less time and led to lower blood loss compared with the anterior approach.

Anterior debridement and interbody fusion can effectively reveal TB lesions, completely remove lesions, and reconstruct the damaged column [8] and is the gold standard for treating spinal TB [9]. In addition, the lack of adhesion of MTB on titanium instruments in vitro prompts spinal internal fixation, which has been proven to be clinically effective and shows excellent radiological performance for kyphosis correction [10, 11]. Dai et al. [12] conducted a prospective study on 39 spinal TB patients who had undergone anterior debridement, autogenous bone grafting and instrumentation, and found that spinal fixation was a safe and effective method of treatment. In a study conducted on 62 spinal TB patients, Obaid-Ur-Rahman et al. [13] reported that anterior debridement with internal fixation resulted in better kyphosis correction and maintenance. However, anterior debridement interbody fusion and fixation have some inherent flaws, such as disturbance to the gastrointestinal tract, complex anatomical structures, large range of exposure, high risk of injury to neural, vascular, and urinary tissue, as well as poor strength of vertebral fixation [14].

Posterior pedicle fixation system is a three-dimensional internal fixation technique that increases stability and corrects spine deformity. In fact, anterior debridement, interbody graft, and posterior pedicle fixation are ideal for spinal TB since it does not damage normal posterior anatomical structures, clearly demarcates the infected area, and can achieve a good radiological result of kyphosis correction [15]. Mukhtar et al. [16] and Talu et al. [17] reported that anterior radical debridement, strut graft fusion and posterior instrumentation are feasible and effective methods for treating TB lesions and achieving long-term correction of kyphosis deformity. A combined anterior and posterior approach is only suitable for spinal TB cases with extensive or fluid paravertebral abscess pus or severely damaged spine stability, since most cases can be treated using a single anterior or posterior surgical approach [18, 19].

Studies have increasingly shown that thoracic and lumbar spinal TB can be successfully treated using a single posterior approach. Yu et al. [20] studied 28 elderly patients with lumbar TB who underwent posterior transforaminal lumbar debridement, interbody fusion, and instrumentation, and found that the operation led to significant ODI improvement and a decrease in the kyphosis angle, although a 2.0° kyphosis correction loss was observed during the follow-up period. Xu et al. [21] found that single posterior debridement, compact bone grafting, and pedicle fixation significantly improved neurological deficit, ODI and led to steady kyphosis correction in 32 patients with mono-segmental lumbar TB and vertebral body damage not more than 2/3rd of the column height. Zhang et al. [22] retrospectively compared a posterior-only, anterior-only, and combined anterior and posterior approaches for spinal TB cases and found that the posterior-only approach was safer and less invasive, compared with the other two approaches. In contrast, Hassan et al. [23] found that a single posterior approach took longer and led to greater blood loss, although it significantly improved kyphotic angle correction and reduced angle loss. This discrepancy of results for operation time and blood loss could be due to various reasons. First, anatomical features are more complex in the anterior approach, especially if the lesion spreads to the retroperitoneal space, which may aggravate the condition. Second, interbody graft material is retrieved from the anterior iliac, which often prolongs the duration of the operation and increases blood loss. Third, the ligation of transverse vertebral arteries is always intractable during lesion clearance or vertebral fixation.

Our study has several limitations, including the non-prospective and double blinded random design, small sample size, and selection bias of surgical decision, which may have affected our conclusions. Therefore, our findings need to be validated using a larger cohort.

## Conclusion

Both single anterior and single posterior approaches of debridement, interbody fusion and fixation are effective for mono-segment lumbar TB patients, although the single posterior approach is of a shorter duration and results in less blood loss.
